# Surface Modification of PLLA, PTFE and PVDF with Extreme Ultraviolet (EUV) to Enhance Cell Adhesion

**DOI:** 10.3390/ijms21249679

**Published:** 2020-12-18

**Authors:** Adam Lech, Beata A. Butruk-Raszeja, Tomasz Ciach, Krystyna Lawniczak-Jablonska, Piotr Kuzmiuk, Andrzej Bartnik, Przemyslaw Wachulak, Henryk Fiedorowicz

**Affiliations:** 1 Department of Biotechnology and Bioprocess Engineering, Warsaw University of Technology, Warynskiego 1, 00-645 Warsaw, Poland; adam.lech@wat.edu.pl (A.L.); tomasz.ciach@pw.edu.pl (T.C.); 2 Institute of Optoelectronics, Military University of Technology, Kaliskiego 2, 00-908 Warsaw, Poland; andrzej.bartnik@wat.edu.pl (A.B.); wachulak@gmail.com (P.W.); henryk.fiedorowicz@wat.edu.pl (H.F.); 3 Institute of Physics, Polish Academy of Sciences, Aleja Lotnikow 32/46, 02-668 Warsaw, Poland; jablo@ifpan.edu.pl (K.L.-J.); kuzmiuk@ifpan.edu.pl (P.K.)

**Keywords:** extreme ultraviolet, surface modification, surface biocompatibility, cell adhesion, PTFE, PLLA, PVDF

## Abstract

Recently, extreme ultraviolet (EUV) radiation has been increasingly used to modify polymers. Properties such as the extremely short absorption lengths in polymers and the very strong interaction of EUV photons with materials may play a key role in achieving new biomaterials. The purpose of the study was to examine the impact of EUV radiation on cell adhesion to the surface of modified polymers that are widely used in medicine: poly(tetrafluoroethylene) (PTFE), poly (vinylidene fluoride) (PVDF), and poly-L-(lactic acid) (PLLA). After EUV surface modification, which has been performed using a home-made laboratory system, changes in surface wettability, morphology, chemical composition and cell adhesion polymers were analyzed. For each of the three polymers, the EUV radiation differently effects the process of endothelial cell adhesion, dependent of the parameters applied in the modification process. In the case of PVDF and PTFE, higher cell number and cellular coverage were obtained after EUV radiation with oxygen. In the case of PLLA, better results were obtained for EUV modification with nitrogen. For all three polymers tested, significant improvements in endothelial cell adhesion after EUV modification have been demonstrated.

## 1. Introduction

Controlling and enhancing the biocompatibility of biomaterials remains a huge challenge in medicine and biomedical engineering. The most important area of the implant or medical device is its surface, the interface between the living organism and the lifeless synthetic product. Processes that happen in this area determine the success of the medical procedure and frequently the survivor of the patient. That is why a scientific effort is focused on searching for more effective techniques of surface modification. Ideally, these techniques should change the properties of the surface without affecting the mechanical properties of the base material. Unfortunately, some conventional techniques, based on photochemical and photophysical processes induced by UV radiation may have undesired effects connected with the penetration of this radiation in polymers [1].

Extreme ultraviolet (EUV) covers a wavelength range of 10–121 nm [2]. This radiation is of particular interest to the modification of polymers due to extremely short absorption lengths in polymers and therefore very strong interaction of EUV photons with materials. EUV photons have a limited depth of penetration into polymers (less than 100 nm). Therefore, the modification of their physical and chemical properties will be limited to the top layer only, leaving the properties of the deeper layers unchanged. The use of EUV can solve the problem of material degradation that occurs with other modification techniques based on UV radiation. Moreover, strong absorption of EUV photons in low-density media makes it possible to perform a photochemical transformation of polymer surfaces using irradiation of material in reactive ambient gases [1].

The use of EUV radiation for modification of polymers is not so popular due to the lack of commercially available laboratory sources of EUV radiation. EUV radiation can be produced by large devices such as synchrotrons or free-electron lasers, which ensure high intensity and consistency of the EUV beam, however, the very high cost of these devices and the limited access results in this source being rarely used in studies for polymer surface modification. An efficient source of EUV radiation is laser plasma produced as a result of the interaction of nanosecond laser pulses with matter. Laser plasma EUV sources are considered for the use in the next generation nanolithography for mass production of integrated circuits [2,3]. Application of a compact laser-plasma EUV source for surface micro-structuring of various polymers with EUV radiation and modification its physicochemical parameters were demonstrated [4,5,6]. It was found that Chinese hamster ovary (CHO) cells show good adhesion and align along an oriented wall- and ripple-type microstructures on PET surfaces produced by the EUV irradiation [7]. These preliminary studies on surface modification of polymers for biocompatibility control via exposure to EUV radiation have been summarized in the review article [8].

Poly(tetrafluoroethylene) (PTFE) is a linear polymer with a repeating monomer structure—[CF_2_CF_2_]_n_–. The polymer is characterized by high viscosity and high molecular weight, which limits the crystallization process, giving it appropriate mechanical properties [9]. In medicine, PTFE has found application as a neovascularization reducing agent [10]. PTFE is also used as a release agent in plastic surgery [11]. These materials prevent tissue adhesion by providing a physical barrier between the injured site and adjacent tissues. Originally PTFE was used in an unmodified form [11], but the development of bacterial film on the suture line and the negative reaction of the body to the implantation after a long-time limited application of PTFE [12]. Currently, this polymer is used in various modified forms to be both antiadhesive and non-toxic to cells [13]. In addition to the above-mentioned PTFE applications, further research is being conducted on the use of this polymer in medicine, including dentistry, as a filling of bone defects or covering of various medical devices. The properties of PTFE were compared with materials currently used in dental techniques, including porcelain, gold alloy, composite resin, and zirconium oxide (ZrO_2_). The achieved results showed that PTFE possesses mechanical properties comparable with other materials tested, however, the bacterial adhesion was the lowest. Unfortunately, the main problem associated with the use of PTFE as an implant in dentistry is the bonding of the polymer to the tooth surface due to its low adhesion properties, so there is a need to modify the polymer surface [14].

Poly(vinylidene fluoride) (PVDF) is a linear, semi-crystalline polymer with a repeatable structure consisting of monomer units—[CH_2_CF_2_]_n_–. It is most often tested for piezoelectric and pyroelectric properties because of its complex polymorphism [9]. PVDF has been used in medicine as a membrane to bind proteins on its surface. Such a film must have the appropriate structure and properties to ensure the binding of proteins due to electrostatic and hydrophobic influences. PVDF exhibits both of these characteristics, being a hydrophobic surface, allowing for strong protein binding, even in unfavorable electrostatic conditions [15]. PVDF is also used as the first generation of drug release stent coatings. Application of PVDF allows to optimize transport of the drug and reduce the irritation caused by the presence of a stent [16]. In recent years researchers are looking for a new medical application for PVDF. The polymer was tested as a scaffold for neural and glial cells [17]. It has been shown that biomaterials containing electrical stimulation (as PVDF due to the mentioned piezoelectric properties) can modulate cell adhesion, proliferation, and differentiation, including nerve cells [18]. The obtained structure would then be used to treat patients with peripheral nerve damage. Of course, the above concept requires additional research, including a comparison of nerve cell growth on the surface of modified and unmodified PVDF [19].

Poly-L-(lactic acid) (PLLA), ([–C(CH_3_)HC(=O)O–]_n_) obtained from lactic acid, has been widely tested for medical applications due to its bioresorbable properties and biocompatibility with the human body [20]. PLLA is widely used in medicine. PLLA fibers, due to their long durability, have been used in the reconstruction of damaged cruciate ligaments and tendons, in two ways: as screws used to bind soft tissues in bone drilling sites and as suture anchors that fix soft tissues to bone surfaces [21]. PLLA is also used as a biodegradable scaffold that replaces stents to treat damaged blood vessels. Stents made from PLLA allows blood vessels to regain its physiological function after complete scaffold degradation [16]. Finally, PLLA is used in surgery as a barrier to prevent the adhesion of adjacent tissues to each other. Usually, after surgeries, the additional fibrous tissue is formed and causes individual organs to adhere to each other. This can lead to severe complications such as small bowel obstruction, chronic abdominal pain, and even infertility. To prevent this phenomenon, a mechanical barrier with antiadhesive properties is used. There are several commercially manufactured barriers, including PLLA (CardioWrap^®^ and SurgiWrap^®^ MAST Biosurgeries USA Inc, San Diego, CA, USA; REPEL CV^®^, SyntheMed Inc, Iselin, NJ, USA), for which a clinically reduced number of pathological adhesions have been confirmed [12].

In the study, we present investigations on surface modification of selected polymers with EUV. The change of basic physicochemical properties of the modified polymer surfaces (morphology, wettability, chemical composition) was analyzed. In the second stage of work, adhesion of human endothelial cells during 72 h of culture was monitored. We hypothesized that the use of EUV radiation for modification of those polymers changes the morphology and chemical composition of the surfaces, which, in turn, affects the number and morphology of surface-adhered cells.

## 2. Results

### 2.1. Wettability

The results of contact angle measurements are summarized in Table 1. When analyzing the contact angle values (CA) obtained for PVDF, it can be seen that both EUV radiation and the presence of reactive gas are needed to obtain a hydrophilic surface. EUV radiation alone not only does not decrease the CA values but even increases (CA = 102.4ᵒ for PVDF_50 vs. CA = 91.1ᵒ for control PVDF). Comparing the wettability results for materials modified with reactive gas (nitrogen or oxygen), two additional relationships can be observed: a lower wettability was obtained for a longer valve opening time (PVDF_50_N400 = 83.3ᵒ vs. PVDF_50_N200 = 98.3ᵒ, PVDF_50_O400 = 70.2ᵒ vs. PVDF_50_O200 = 87.9ᵒ); also, the lower CA value was obtained for modification process performed with oxygen as compared to nitrogen (PVDF_N200 = 98.3ᵒ vs. PVDF_O200 = 87.9ᵒ, PVDF_N400 = 83.ᵒ vs. PVDF_O400 = 70.2ᵒ). However, these are not statistically significant differences. It can, therefore, be concluded that the wettability of PVDF after modification does not change significantly.

Other results were obtained for PTFE. Surprisingly, for this polymer, the lowest contact angle was obtained for a sample exposed to EUV radiation without reactive gas (PTFE_50 = 82.8ᵒ). If additional gas is used in the modification process, values of CA are higher for all tested variant (PTFE_50_N200 = 113.6ᵒ, PTFE_50_N400 = 109.3ᵒ, PTFE_50_O200 = 106.2ᵒ, PTFE_50_O400 = 96.3ᵒ). At the same time, the values are slightly lower compared to non-modified material. The lowest CA value was obtained for PTFE_50_O400 = 96.3ᵒ. Still, the value was higher compared to polymer modified without reactive gas.

The relationships obtained for PLLA are similar to those obtained for PVDF. After applying E-UV radiation, without additional reactive gas, the hydrophilicity of the material was reduced; CA values were 76.1ᵒ and 82.8ᵒ, for control PLLA and PLLA_50 respectively. After the application of nitrogen, the contact angle value remained at a level close to that for PLLA_50 material (PLLA_50_N200 = 82.1ᵒ, PLLA_50_N400 = 77.3ᵒ). A significant decrease in the contact angle was obtained when applying oxygen during the modification process; also, the decrease was higher for longer exposure time (PLLA_50_O400 = 53.7ᵒ, PLLA_50_O200 = 71ᵒ).

Comparing all three polymers, it can be seen that the lowest CA values were obtained for PLLA, this applies to all analyzed surface variants.

### 2.2. Chemical Composition Analysis

Table 2 presents the atomic percentage of elements in each surface variant of PVDF and the chemical bonds of C. First of all, a decrease in the fluorine content after EUV irradiation is visible for all surface variants. As expected, the fluorine content is the smaller the longer contact time with the gas. Interesting results are obtained for surfaces modified with nitrogen exposure: only a longer opening time of the valve (higher gas pressure) (PVDF_50_N400) caused the element to join the structure, while its amount is still small (8.2%). As expected, the amount of built-in oxygen is significantly higher in the case of surfaces modified in oxygen.

**Figure 1:**
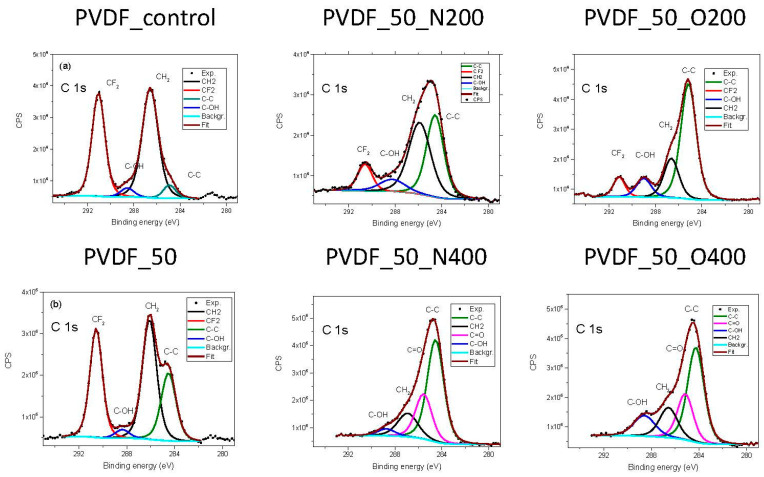
XPS spectra of C1s recorded for each PVDF surface variant.

In the literature, you can find the C1s spectrum for PVDF with two peaks, that represents two bonds: –CF_2_– (with binding energy value of 290.5 eV) and –CH_2_– (with a value of 285.8 eV) [22]. Additionally, we have recorded two small peaks attributed to environmental contamination. Analysis of the C1s spectra of investigated samples (Figure 1) confirms the previously described results: there is a degradation of the carbon-fluorine bond (decrease in the number of –CF2– bonds), while new carbon bonds with oxygen appears. The longer valve opening time (either for nitrogen or oxygen) the more gas atoms are presented in the surface. In the case of PVDF_50_N400 nitrogen appeared in the chemical composition. Simultaneously, a peak with BE 285.6 eV appeared in C1s spectrum. This peak appeared also in the spectrum recorded for PVDF_50_O400, for this variant no nitrogen was present in the chemical composition. The binding energy of around 285 eV is similar for C-N bond and a double bond of C with O. Thus, it might indicate a formation of C–N bond in the case of PVDF_50_N400 and/or C=O in the case of PVDF_50_O400.

As in the case of PVDF, the content of elements was also examined for PTFE and the analysis of narrow spectra for carbon was performed (Table 3). Unlike PVDF, after EUV irradiation, the amount of fluorine remained at a similar level for all variants of surface modification (the maximum difference between the highest and the lowest value is 2.6%). However, a small amount of oxygen was built into the structures of PTFE. Surprisingly, the percentage of oxygen was slightly higher for shorter opening times (3.6% for PTFE_50_O200 vs. 1.4% for PTFE_50_O400). No nitrogen was found in the structure, also for samples modified with nitrogen.

**Figure 2:**
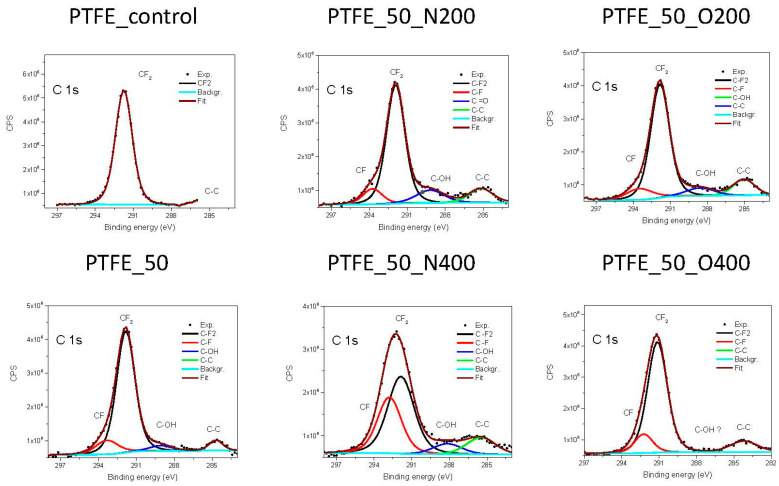
XPS spectra of C1s recorded for each PTFE surface variant.

The chemical structure of PTFE consists of –CF_2_– bonds, which have a binding energy of 292.5 eV [23]. The analysis of narrow carbon 1s spectra (Figure 2) shows that after EUV irradiation a decrease in bonds –CF_2_– appears. At the same time, new –CF– bonds are formed. The longer valve opening time (either for nitrogen or oxygen) the more of –CF– bonds are created at the surface. In a case of two surface variant, PTFE_50, and PTFE_50_N200, residual bonds of carbon with oxygen (treated as impurities) appears.

In the case of PLLA (Table 4), a decrease in the amount of oxygen to carbon is obtained for every surface modification variant, therefore the O 1s spectra were also analyzed. The 2:1 ratio of carbon to oxygen increases to 3:1 after EUV radiation, which means that there are three carbon atoms per oxygen atom, i.e., an additional carbon atom appears with oxygen as a reactive gas. The use of reactive gases changes this ratio in the error limit only, besides the case of longer nitrogen valve opening time, where the slowdown of this process was observed. The ratio of C to O is in this case 3:2. The only change is observed with a longer valve opening time and the uses of nitrogen as reactive gas (the ratio is approximately 3:7). No nitrogen was found in the structure, also for samples modified under a nitrogen atmosphere.

**Figure 3:**
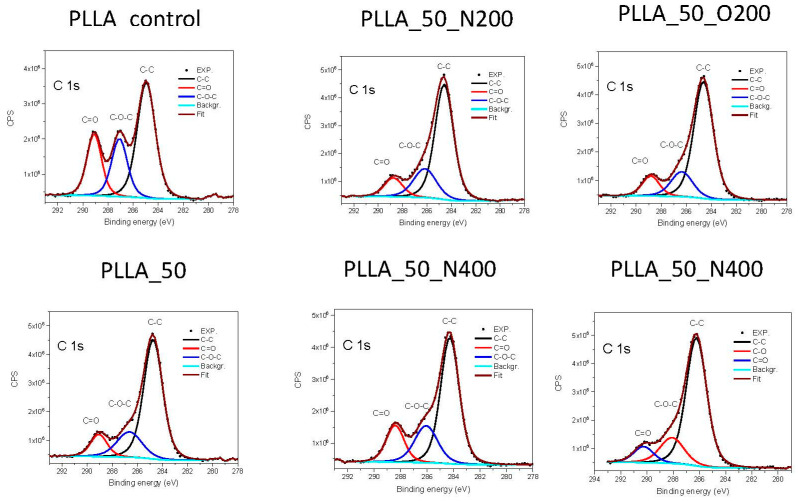
XPS spectra of C1s recorded for each PLLA surface variant.

Analysis of narrow C 1s (Figure 3) and O 1s spectra for PLLA, revealed the presence of three types C of bonds with the binding energy indicated in brackets: –C–C– (285 eV), –C–O–C– (287 eV) and –C=O– (289 eV) and two types of O bonds: –O=C– (532 eV) and –O–C– (533.4 eV) [24]. The most visible change is in the amount of bonds –C=O– at the site of C and –O–C– at the site of O, which has partially degraded as a result of the radiation. Therefore, the –O–C– bonds are broken and additional C is attached to the polymer surface. The use of nitrogen with longer valve opening time (PLLA_50_N400) slightly slows down this process, although the quantity of this bond is still smaller compared to the original surface. The use of oxygen, in turn, causes further degradation of this bond. The longer valve opening time for oxygen also reduces the amount of –C=O– bond but not that much the –O–C– bond.

### 2.3. Surface Morphology

**Figure 4:**
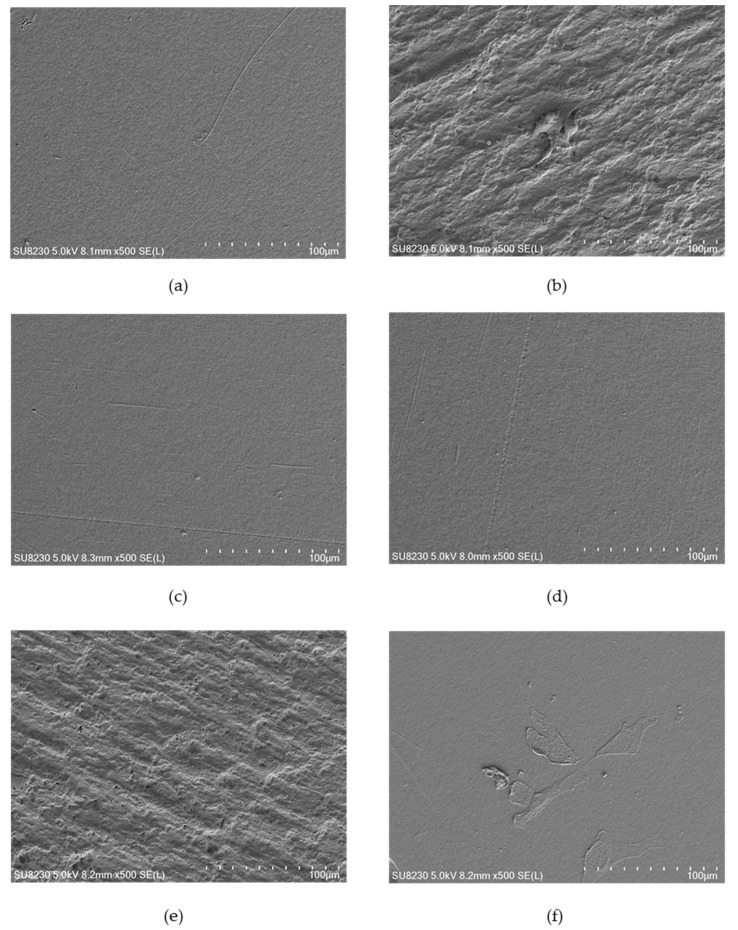
EUV modified PVDF with surface-adhered cell safter 24 h of culture: (**a**) PVDF_control, (**b**) PVDF_50, (**c**) PVDF_50_N200, (**d**) PVDF_50_N400, (**e**) PVDF_50_O200 and (**f**) PVDF_50_O400. Magnification = 500×.

**Figure 5:**
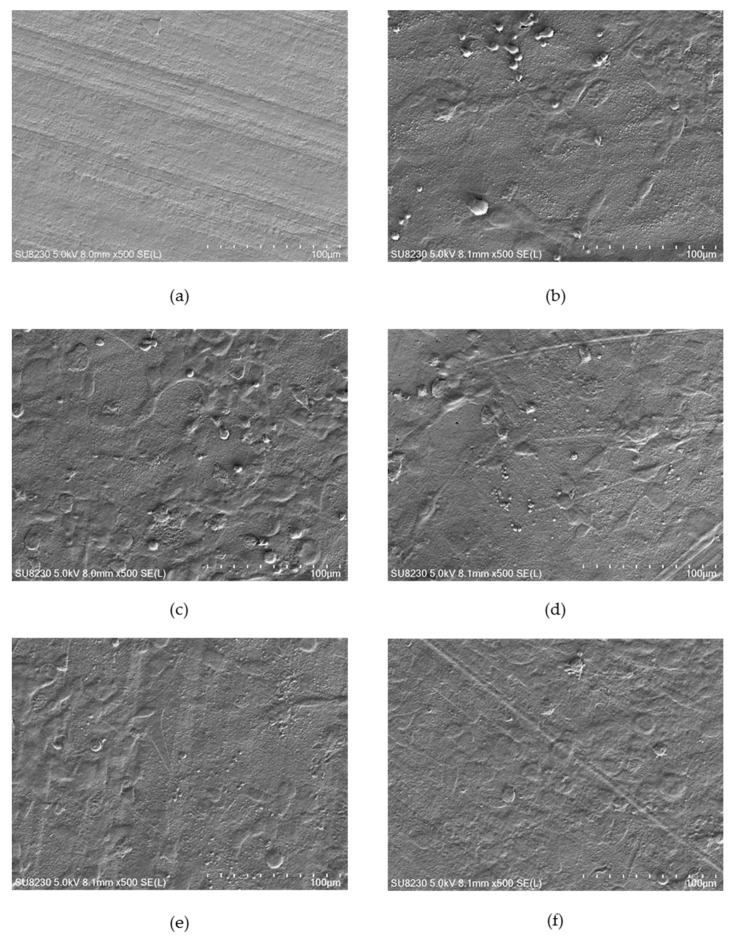
EUV modified PTFE with surface-adhered cells after 24 h of culture: (**a**) PTFE_control, (**b**) PTFE_50, (**c**) PTFE_50_N200, (**d**) PTFE_50_N400, (**e**) PTFE_50_O200 and (**f**) PTFE_50_O400. Magnification = 500×.

**Figure 6:**
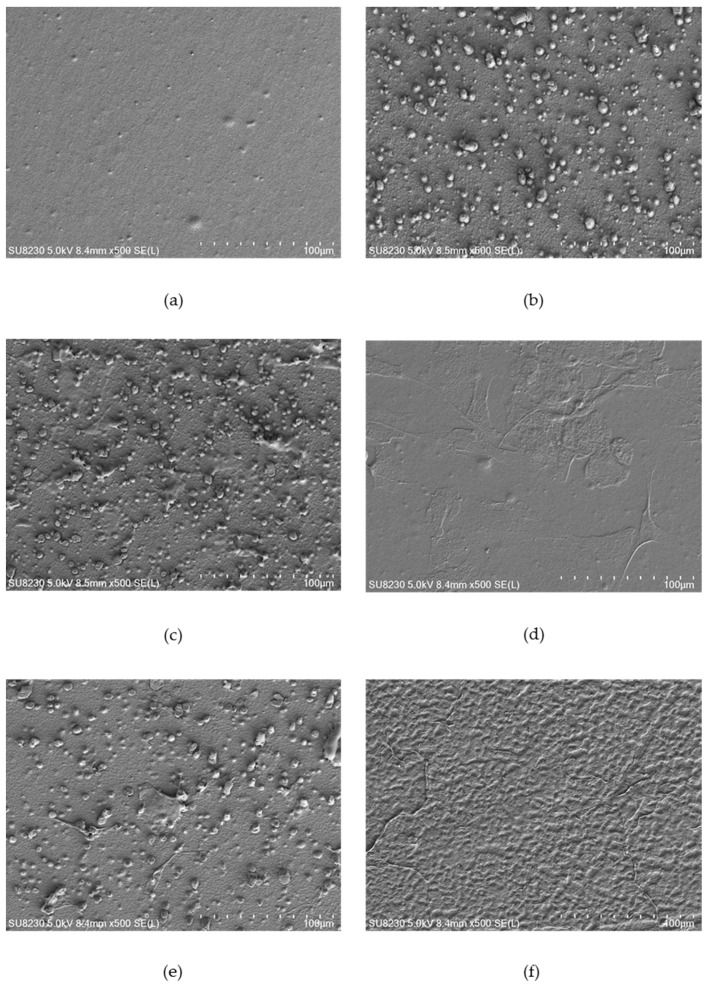
EUV modified PLLA with surface-adhered cells after 24 h of culture: (**a**) PLLA_control, (**b**) PLLA_50, (**c**) PLLA_50_N200, (**d**) PLLA_50_N400, (**e**) PLLA_50_O200 and (**f**) PLLA_50_O400. Magnification = 500×.

Figure 4, Figure 5 and Figure 6 show selected images of modified surfaces with cells adhered to the polymers after 24 h of culture. Analysis of the images allows determining the impact of EUV irradiation on PVDF (Figure 4), PTFE (Figure 5) and PLLA (Figure 6) surfaces.

The PVDF control presents a smooth surface with no visible surface-adhered cells. On PVDF_50 an increase in surface roughness is visible. Single cells with flattened morphology were also observed. Surprisingly, EUV radiation with nitrogen for both valve opening times does not bring about any changes in surface roughness compared to the control sample. Additionally, there were no cells observed. Modification with oxygen gave different results depending on the valve opening time. For a shorter time, (200 ms) morphology of the surface was similar to the one presented on PVDF_50. Surprisingly, surface modified with oxygen with the longer valve opening time (400 ms) did not show any changes compared to the control surface. However, in contrast to the control surface, there were few surface-adhered cells with flattened morphology observed.

The surface of the control PTFE presented a slightly rough morphology with no surface-adhered cells. After EUV modification, an increase in surface roughness can be observed for all modification variants. Additionally, a significant increase in the number of surface-adhered cells was observed for all modification variants. No significant differences in morphology and the number of surface-adhered cells were observed between the modification variants. For all EUV-modified variant areas with characteristics, the pyramidal structure appeared. However, in areas where these structures were present and in areas without these structures, the number of cells was similar.

The control PLLA surface presented a slightly rough morphology with no surface-adhered cells. Microscopic analysis of PLLA after EUV modification revealed a significant increase in roughness for three modification variants: PLLA_50, PLLA_50_N200, and PLLA_50_0200. Similar to PTFE, a characteristic pyramidal structure appeared, which is covered with surface-adhered cells. Interestingly, PLLA_50_N400 presented morphology similar to the control surface–no increase in roughness nor pyramidal structures were observed. However, there were many surface-adhered, flattened cells presented. A similar result was obtained for surface modified with oxygen with longer valve opening time, PLLA_50_O400. For that sample surface roughness was slightly higher compared to PLLA_50_N400, but significantly lower compared to other EUV-modified surfaces. Nevertheless, the surface was covered with some strongly flattened, surface-adhered cells.

### 2.4. Cell Adhesion

**Figure 7:**
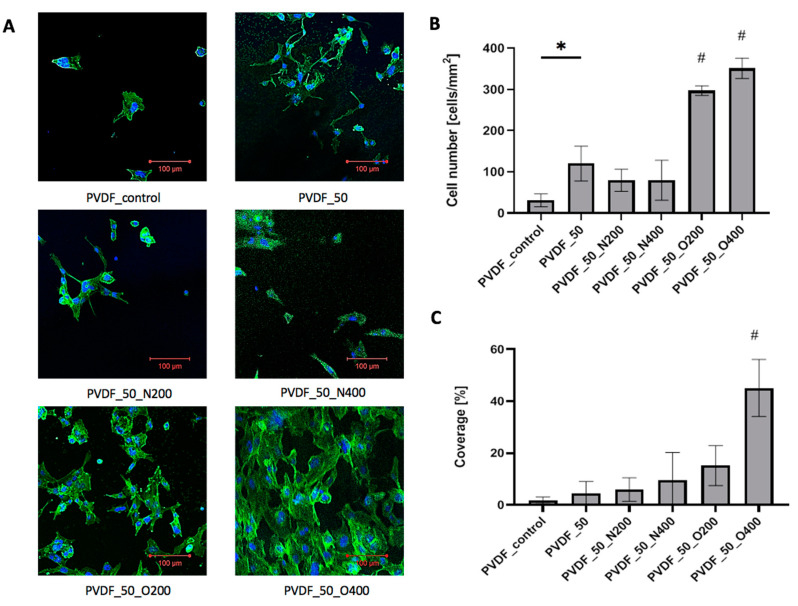
(**A**) Cells adhered to PVDF surfaces after 72 h of culture. (**B**) The number of cells adhered to PVDF surfaces after 72 h of culture. A significantly low number of cells adhered can be observed in the case of PVDF_control compared to PVDF_50 (* *p* = 0.0084) and a high number of cells adhered can be observed in the case of PVDF_50_O200/PVDF_50_O400 compared to other PVDF variants (# *p* < 0.05). (**C**) The percentage of cell coverage obtained for PVDF after 72 h of culture. # For *p* < 0.05 vs. each other variant. Significantly high cell coverage can be observed in the case of PVDF_50_O400 compared to other PVDF variants (# *p* < 0.05).

Cells adhered to control PVDF presented proper morphology, however, the cell-surface contact area was small (Figure 7A). The number of surface-adhered cells was low (31.06 cells/mm^2^) (Figure 7B). The overall cell coverage obtained after 72 h of culture was less than 2% (1.8%) (Figure 7C). After EUV surface modification (without reactive gas) the number of surface-adhered cells increased (120.20 cells/mm^2^). At the same time, the cell-surface contact area remained small-cell coverage after 72 h of culture equaled 4.5%. EUV modification with nitrogen gave similar results of cell number—the values were 79.68 cells/mm^2^ for shorter valve opening time (PVDF_50_N200) and 79.68 cells/mm^2^ for longer valve opening time (PVDF_50_N400). However, the microscopic observation revealed that after modification with nitrogen the cell-surface contact area increased—the cells were more flattened compared to PVDF_50. The cell coverage was 6.0% for PVDF_50_N200 and 9.5% for PVDF_50_N400. EUV modification with oxygen gave the best result of cell number and cell coverage. Cell number significantly increased for both variants: PVDF_O200 (297.11 cells/mm^2^) and PVDF_O400 (351.13 cells/mm^2^). The difference between both variants was statistically significant—a higher cell number was observed for longer valve opening time (400 ms). A similar relation was observed for cell coverage—both variants modified with oxygen presented higher cell coverage compared to other surface variants. Both PVDF_O200 and PVDF_O400, were covered by a high number of strongly flattened cells—large cell-surface contact area was observed. The coverage values for PVDF_O200 was 15.2%. In the case of PVDF_50_O400, the coverage rate after 72 h of culture was the highest and exceeded 40% (45.1%).

**Figure 8:**
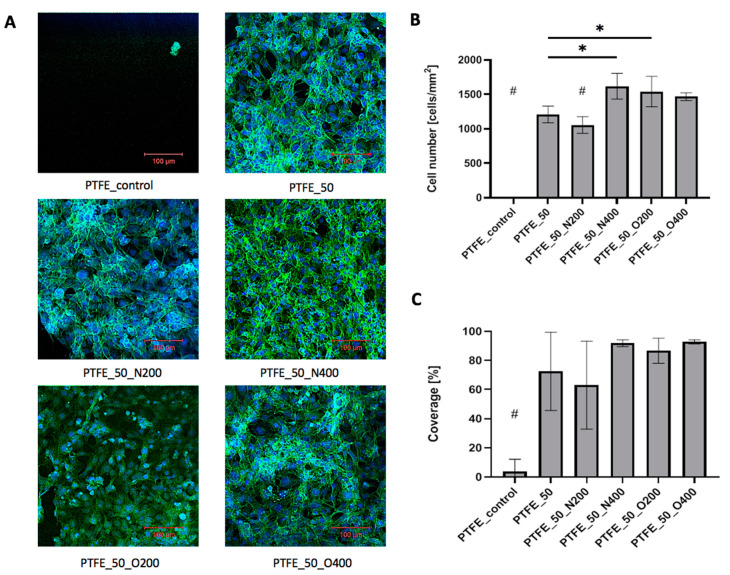
(**A**) Cells adhered to PTFE after 72 h of culture. (**B**) Number of cells adhered to PTFE surfaces after 72 h of culture. A significantly low number of cells adhered can be observed in the cases of: PTFE_control/PTFE_N200 compared to other PTFE variants (# *p* < 0.05) and PTFE_50 compared to PTFE_50_N400 (* *p* = 0.006) and PTFE_50_O200 (* *p* = 0.032). (**C**) The percentage of cell coverage obtained for PTFE after 72 h of culture. # for *p* < 0.05 vs. each other variant. Ssignificantly low cell coverage can be observed in the case of PTFE_control compared to other PTFE variants (# *p* < 0.05).

In the case of PTFE surfaces, after 72 h of culture, control PTFE was covered with single, loosely adhered, round cells (Figure 8A). The difference in cellular adhesion between the control surface and EUV modified surfaces was visible. Each EUV modified surface presented a significantly higher number of adhered cells (Figure 8B) and cellular coverage (Figure 8C). The very high cell number values were obtained for the oxygen-modified variants: PTFE_O200 (1542.28 cells/mm^2^) and PTFE_O400 (1466.66 cells/mm^2^). A similar result was obtained for surface modified with nitrogen with longer valve opening time: PTFE_N400 (1619.26 cells/mm^2^). As mentioned EUV modified surfaces exhibited high cellular coverage, significantly higher compared to control PTFE (cellular coverage = 4.1%). For PTFE_50 and PTFE_50_N200 the cellular coverage exceeded 60% (PTFE_50 = 72.6%, PTFE_50_N200 = 63.0%). Higher values were obtained for materials modified with oxygen—cellular coverage equaled 86.7% for PTFE_O200 and 92.9% for PTFE_O400. Similar result was obtained for PTFE_50_N400 (cellular coverage = 92.0%). Microscopic analysis showed that all EUV modified samples were coated with cells presenting proper morphology.

**Figure 9:**
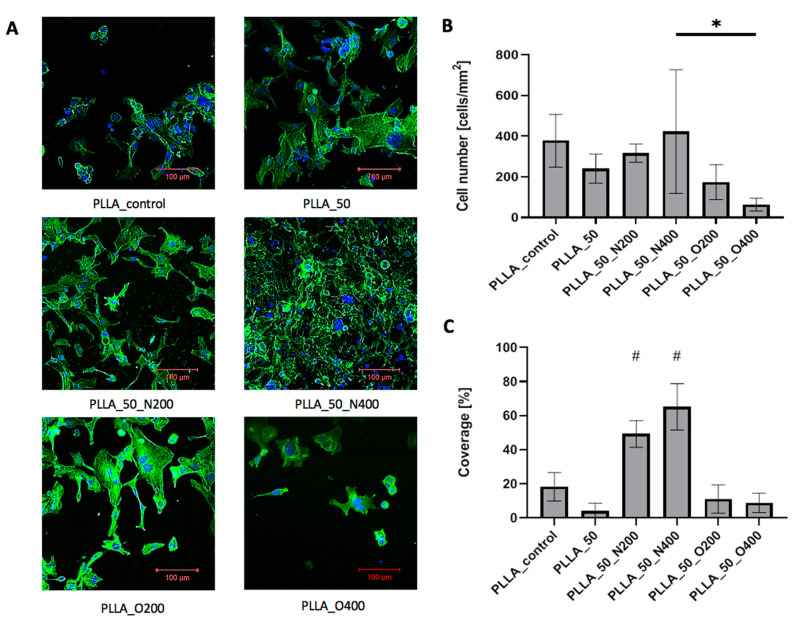
(**A**) Cells adhered to PLLA surfaces after 72 h of culture. (**B**) The number of cells adhered to PLLA surfaces after 72 h of culture. A significantly low number of cells adhered can be observed in the case of PLLA_50_O400 compared to PLLA_50_N400 (* *p* = 0.025). (**C**) The percentage of cell coverage obtained for PLLA after 72 h of culture. # for *p* < 0.05 vs. each other variant. A significantly high number of cells adhered can be observed in the case of PLLA_50_N200 and PLLA_50_N400 compared to other PLLA variants (# *p* < 0.05).

In the case of PLLA, all samples were coated with cells presenting proper morphology (Figure 9A). The influence of EUV radiation on cell adhesion was less visible. Cell number (Figure 9B) was similar for control PLLA (378.14 cells/mm^2^), PLLA_50 (240.39 cells/mm^2^), PLLA_50_N200 (316.14 cells/mm2) and PLLA_50_N400 (422.71 cells/mm^2^). Values obtained for samples modified with oxygen were lower: 174.22 cells/mm^2^ for PLLA_50_O200 and 63.47 cells/mm^2^ for PLLA_50_O400. Cellular coverage (Figure 9C) equaled 18.2% for control PLLA. Surprisingly again, the values were smaller for samples modified without reactive gas: 4% for PLLA_50 and samples modified with oxygen: 11.1% for PLLA_50_O200 and 8.8% for PLLA_50_O400. The higher cellular coverage was obtained for samples modified with nitrogen: 49.3% for PLLA_50_N200 and 65.2% for PLLA_50_N400.

## 3. Discussion

In the case of PVDF, wettability analysis showed that the exposure of the material to EUV radiation without additional reactive gas increases the hydrophobicity of the surface. When nitrogen or oxygen was present during the EUV irradiation, the wettability of the surface increased. Additionally, the hydrophilicity increased for longer valve opening time. Surprisingly, the change in wettability did not correspond with the change in surface morphology. Only for surfaces modified without reactive gas and for oxygen-modified with 200 ms valve opening time the increase in surface roughness was visible. In the rest of the EUV modified surface variants, the morphology was comparable to the control material. The biggest change in chemical composition was observed in the fluorine content. For the control surface the percentage of fluorine was 47.4%, after EUV irradiation (without additional reactive gasses) it dropped below 40%. When oxygen or nitrogen was used together with EUV radiation, the fluorine content dropped even more (<30%). In its place, nitrogen and oxygen were built in. Surprisingly, oxygen was present even in variants modified with the use of nitrogen. This oxygen probably comes from the residual gas present in the evacuated chamber. However, the change in the chemical composition of the PVDF surface is evident. Additionally, the EUV irradiation with the addition of oxygen has the strongest effect of cell adhesion. Both, the cell number and the cell surface were the biggest in the case of surfaces modified with oxygen. This effect is consistent with the results of chemical composition analysis (the highest percentage of oxygen) and wettability analysis (high hydrophilicity).

The effect of EUV irradiation on the process of cell adhesion to PTFE surfaces was very clear. Unmodified PTFE was an unfavorable material for cell growth. After 72 h of culture, the PTFE surface was coated with few, loosely adhered, spherical cells. On the contrary, all EUV-modified surfaces strongly supported cell adhesion and were coated with several well-adhered, flattened cells. After 72 h of culture, the cellular coverage exceeded 60% for all EUV-modified variants. The use of oxygen as reactive gas favors cell proliferation. In the case of nitrogen, the cell adhesion was smaller compared to variants modify with oxygen, however, the number of cells was still significantly higher compared to the control material. Chemical analysis revealed no significant changes in elements content after EUV treatment. In contrast to PVDF, the stability of elements content in PTFE is visible—the maximum difference in fluorine content between the variants is 2.6%. However, there was a change in fluorine chemical bonding. A gradual converting of –CF_2_– to –CF_3_ bond was observed. This change proved the interaction of EUV radiation with the structure of the material. In terms of surface wettability, the biggest change was observed for surfaces modified with EUV without reactive gas—in this case, the obtained surfaces were the most hydrophilic. Application of reactive gas (either nitrogen or oxygen), slightly reduced this effect, however, the contact angle values were still lower compared to control PTFE. The EUV irradiation influence was also visible in the morphology change—all EUV-modified variants exhibit increased surface roughness.

In the case of PLLA, the effect of EUV modification was also clearly visible. After EUV irradiation the contact angle values changed—depending on the type of reactive gas, this value decreased (for nitrogen) and increased (for oxygen and modification without reactive gas). EUV irradiation also causes changes in the chemical structure, reducing the amount of oxygen, the level of reduction was smallest for N longer opening time. The change in surface morphology was uneven. The significant impact of EUV radiation was observed for surfaces modified without reactive gas as well as surfaces modified with oxygen and nitrogen with shorter valve opening time. For those surfaces where the roughness increased noticeably, many characteristic pyramidal structures appeared. Surprisingly, two variants—surfaces modified with nitrogen and oxygen with longer valve opening time (400 ms)—did not show a similar change in morphology. The morphology change was not correlated with an increase in cell adhesion. Additionally, the effect of EUV modification was not clear. Higher cell coverage was obtained for surfaces modified with nitrogen and the highest cell number for nitrogen longer opening time, where the smallest reduction of oxygen was found. The influence of oxygen was negative—the number of surface adhered cells did not increase compared to control PLLA, although the cell surface contact area for individual cells was visibly bigger.

So far, not many studies are examining the impact of EUV surface modification on polymer biocompatibility and cell adhesion. In our previous studies, we examined EUV-modified PTFE. We demonstrated the increased fibroblast adhesion to the PTFE surface after EUV modification. The number of surface-adhered cells was correlated with the number of laser shots used to modify the surface [8]. Reisigner et al. [7] demonstrated good cell adhesion to EUV modified PET.

The effects of surface modification with EUV radiation can be compared with results obtained for UV radiation. Girardeaux et al. (1996) [25] investigated the influence of UV radiation of PTFE performed under vacuum, in atmospheric air, and additional nitrogen and in oxygen presence. While no changes were shown in the first three cases, a decrease in the F/O ratio from 2 to 1.13–1.30 was observed for oxygen presence. The share of chemical bonds is also changing: –CF_2_– is still the most intense one, but new bonds with fluorine are being created next to it, e.g., –CF_3_. The influence of UV radiation on surface wettability was studied elsewhere [26]. The results indicated a decrease in wettability depending on the length of laser exposure time. It should be noted that the greatest decrease was observed during the first 5 min of surface exposure to the laser beam. The cell density for surfaces modified with UV was compared in Gumpenberger et al. (2003) [27]. The obtained results show a strong positive effect of the UV radiation on cell adhesion. It can be concluded that both EUV and UV modification techniques resulted in a decrease of PTFE wettability. Another common result of both modification techniques is an increase in oxygen content; however, the F/O ratio depends on reactive gas used during radiation. Both UV and EUV radiation resulted in a strong increase in the number of cells adhered to the modified PTFE.

In the case of PVDF, the UV radiation has been shown to strongly influence the surface wettability [28]. UV exposure to PVDF strengthened its hydrophobic properties, the obtained contact angle values were close to 175ᵒ, which corresponds to the values for superhydrophobic surfaces. These results differ from the results obtained for EUV modification. We observed significantly smaller contact angle values (mostly below 100ᵒ). Under the influence of UV radiation, the chemical composition of PVDF has also changed. As a result of exposure, new functional groups, mainly with fluorine and oxygen, appear. At the same time, the percentage of –CF_2_– group has been significantly reduced [28]. Therefore, the results of chemical analyses after a EUV and UV radiation seem to be similar. In both cases, there was a fluorine loss from the material (but for EUV no other fluorine bonds than –CF_2_– were found) and new functional groups with oxygen appeared.

A change in wettability and the chemical composition after UV irradiation was also observed for PLLA. Slepička et al. (2012) [29] (KrF laser, no reaction gas) described a decrease in wettability after UV treatment; the change of contact angle values was influenced by laser power and the number of pulses. In the case of chemical composition, no new bonds were observed, although new peaks appear. However, their chemical composition is the same as for unmodified PLLA (–C–C–, –C–O– and –C=O–), they are also shifted (by about 10 eV) and more intense. The results of cytocompatibility for UV modified PLLA could be also found in the literature. To achieve the evident changes in the results, in addition to laser exposure (KrF, no additional gas), the PLLA surface was also subjected to thermal annealing. The results obtained show a positive effect of UV radiation on the increase in cell viability, although the laser beam must not be too intense. With too much energy density, the process of cell adhesion to the substrate is significantly inhibited [30].

In conclusion, both EUV- and UV-based modification changes the wettability and chemical composition of the studied polymers. However, it should be stressed that EUV radiation is a much less invasive technique that changes only the thin outer surface of the polymer without interfering with its deeper layers. At the same time, the modification is sufficient to achieve a biological effect—increased adhesion of cells to the surface of the material.

## 4. Materials and Methods

### 4.1. Modification of Polymer Surfaces by EUV Radiation

**Figure 10:**
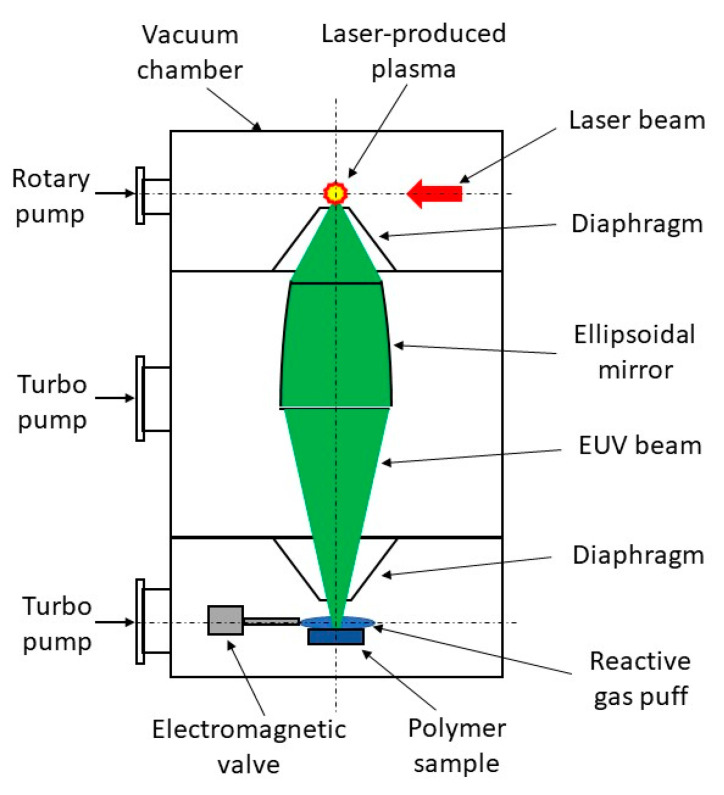
Schematic of the laboratory system for modification of polymer surfaces with extreme ultraviolet (EUV) radiation.

Modification of polymer surfaces with EUV has been performed using a home-made laboratory system developed at the Institute of Optoelectronics MUT. The system is based on a compact laser-plasma EUV source [31]. EUV radiation is produced in a hot plasma generated as a result of irradiation of a double-stream gas puff target with nanosecond laser pulses. The use of the gas puff target instead of a solid target allows efficient generation of EUV radiation without the harmful effect of target debris associated with laser ablation from solids. The schematic of the system for EUV modification of polymers is shown in Figure 10. The EUV system is composed of a vacuum chamber in the form of a vertical column. The chamber is divided into three sections separated with small diaphragms. The use of the diaphragms makes it possible to pump separately each section by oil-free dry and turbo vacuum pumps (differential pumping). In the first upmost section of the chamber, the laser-plasma EUV source based on a double-stream gas puff target is placed. The plasma is generated by irradiating the target with laser pulses from an Nd:YAG laser (NL 303 HT, EKSPLA, Vilnius, Lithuania) with a pulse energy of 0.8 J, pulse duration of 4 ns, operating at 10 Hz repetition rate. EUV radiation emitted from the point-like source in the form of 4 ns pulses is collected with a grazing incidence axisymmetrical ellipsoidal mirror placed in the middle section of the chamber and focused onto a sample mounted in the third bottom section with the use of the computer-controlled x-y-z translation stages. It allows irradiation of samples up to 5 cm wide. The sample section of the chamber can be evacuated to a high-vacuum range (10^−5^ mbar) due to the differential pumping. EUV radiation can be focused to a spot of about 1 mm in diameter with fluency up to 70 mJ/cm^2^ for the xenon gas puff target. The spectrum of EUV radiation in the focus consists of a strong narrow spectral feature with an intensity maximum near 10 nm wavelength and a long-wavelength tail up to 70 nm. The sample section is also equipped with an electromagnetic valve for the injection of reactive gas (high purity nitrogen or oxygen, purity of 99.9992%) onto the sample in the EUV irradiation region. Gas pressure in the EUV irradiation region was changed by changing the valve opening time. The system design and its parameters are described in detail in the article [32]. The preliminary investigations on EUV modification of polymer surfaces for control of biocompatibility using this system have been performed by Ahad et al. [8,33].

In the presented studies the described system was used for surface modification of the selected polymers. The modification parameters for the chosen polymers are given in Table 5. The square area of 5 × 5 mm was modified on every sample. All tested polymers (PTFE, PLLA, PVDF) were purchased from Goodfellow Cambridge Ltd., Huntingdon, UK.

### 4.2. Analysis of Chemical Composition

The chemical composition of materials after EUV-induced modification were analyzed at the Institute of Physics, Polish Academy of Sciences in Warsaw using X-ray photoelectron spectroscopy (XPS). After previous preparation, the samples were measured by the Scienta R4000 analyzer (PREVAC, Rogów, Polnad), using Kα Al radiation without a monochromator. The C, O, F, and N 1s spectra were measured and analyzed using the CasaXPS program.

### 4.3. Wettability

Wettability measurement with a goniometer was carried out determining the angle between the polymer surface and a single drop of water in contact with it. The ADVANCE program used for this purpose generated a straight line identical to the surface of the material in the photo showing the drop on the surface and then recalculated the contact angle of the drop with the surface (static contact angle measurement).

### 4.4. Cell Culture

Human microvascular endothelial cells (HMEC, Lonza, Basel, Switzerland) were maintained and cultured in MCDB medium (Gibco, Thermo Fisher Scientific, Waltham, MA, USA) supplemented with 10% FBS, 1% pen–strep, 10 ng/mL EGF and 10 mM L glutamine) from Gibco. Materials samples were cut to the form of discs (with a diameter 8 mm) and sterilized with a 100 mL solution of 1 mL mixture of penicillin (100 μg/mL), streptomycin (100 U/mL) and 100 μL amphotericin (0,25 μg/mL) for 1 h at 4 °C. After sterilization materials were washed with PBS, placed in 24-well plates, and incubated with supplemented MCDB for 1 h at 37 °C. Next, cells were harvested, seeded on the material, and cultured at 37 °C for 24 and 72 h in a humidified atmosphere with 5% CO_2_.

### 4.5. Surface Morphology

After 24 h of culture, materials were analyzed with a scanning electron microscope (SEM). Samples were removed from the medium, washed with PBS, fixed with 4% paraformaldehyde, dehydrated in graded ethanol/water solutions, and dried at RT. Finally, samples were covered with gold and observed with SEM.

### 4.6. Cells Adhesion

After 72 h of culture, materials were analyzed with a confocal scanning microscope (CLSM). At the end of the culture, samples were removed from the medium, washed with PBS, and fixed with 4% paraformaldehyde. The following staining procedure was applied: samples were rinsed 3 times with PBS, incubated 4 min in 0.2% (*v*/*v*) Triton X-100, rinsed 3 times with PBS, incubated in 0.1% (*w*/*v*) Bovine Serum Albumin solution for 1 h, rinsed 3 times with PBS, incubated with AlexaFluor Phalloidin 488 for 30 min, rinsed 3 times with PBS, incubated with DAPI (4′,6-Diamidino-2-phenylindole dihydrochloride) for 5 min, rinsed 3 times with PBS. Materials were glued to the cover glass with ProLong^®^ Gold Antifade Mountant (ThermoFisher, Willow Creek, CA, USA) and left until analysis.

Every surface variant was analyzed in triplicate, for every sample at least 5 randomly selected spots were depicted. Images were analyzed using ImageJ. Cell number was calculated using Cell Counter plugin and manual counting. Every DAPI-stained nucleus was counted as one cell. The cell-coated area was calculated using single-color image (CLSM images saved in “one channel” mode), by setting the threshold and using the particle analysis feature.

### 4.7. Statistical Analysis

Wettability, cell number, and cellular coverage were expressed as means ± SD. Statistical significance of differences was analyzed using a single-factor analysis of variance (ANOVA) with post hoc Tukey’s test (OriginPRO 8.0, OriginLab Corporation, Northampton, MA, USA). *p*-values of < 0.05 were considered statistically significant.

## 5. Conclusions

In the study, we presented the results of detailed investigations on polymer surface modification for biocompatibility control using EUV radiation produced with a laser-plasma EUV source. Three polymers, widely applied in bioengineering, were modified: PVDF, PTFE, and PLLA. Then, we analyzed the influence of modification process parameters such as the number of EUV pulses, the application of additional reactive gases (oxygen and nitrogen), and reactive gas density controlled by valve opening time on the process of endothelial cells adhesion.

In the case of PVDF, the enhancement in cell adhesion was obtained for some modification variants. The morphology of the surface subjected to the radiation changes, becoming rougher, but only for some variants. Surface wettability changes, depending on the modification variant. EUV irradiation strongly increased the content of oxygen in the surface on all modified surfaces. This increase was higher for variants with oxygen as a reactive gas. For those variants also a significant increase in the number of surface-adhered cells was observed after 72 h of culture. The use of nitrogen as reactive gas does not cause such an evident increase in the number of cells. Nevertheless, a certain increase can be observed, higher for longer valve opening time.

Of all the materials, by far the best results were obtained for PTFE, for which there is a clear increase in cell adhesion for each variant of the modification. The material has stable content of elements. Nevertheless, besides carbon bonded with two fluorine atoms, carbon bonded with three fluorine was detected during the surface modification. Surface wettability only slightly increased after EUV irradiation for all modification variants. Increased roughness of the surface was observed for each modification variant.

The results of studies for PLLA turned out to be the least transparent. The analysis of the chemical composition shows a change in the carbon/oxygen ratio from 2:1 to 3:1 therefore, the reduction of oxygen content was observed. Surface wettability differs depending on the modification variant. The application of nitrogen as reactive gas increased CA values. The surface morphology also changes, but only for variants with short valve opening time and for variant modified without reactive gas. The use of nitrogen had a positive effect on the growth of the cells and for longer nitrogen exposer limited oxygen reduction. The effect of oxygen remains unobvious and needs further investigation.

In conclusion, it has been shown that the modification of the tested polymers with EUV radiation has a significant impact on the cellular adhesion of polymers. Among the analyzed process parameters, the use of nitrogen or oxygen as a reactive gas significantly affects the number of cells and the percentage of cellular coverage. Determining more precise relations between cellular adhesion and the type of reactive gas used in the modification process requires further research.

## Figures and Tables

**Table 1:** Results of contact angle measurements for control polymer (n—number of measurements, *n* = 2) and EUV modified surfaces (*n* = 8). A significantly low wettability can be observed in three cases of: PVDF_50_O400 compared to PVDF_N200 and PVDF_50 (* *p* < 0.05); PTFE_50/PLLA_50_O400 compared to other PTFE/PLLA variants (^&^ *p* < 0.05); PTFE_50_O400 compared to PTFE_control, PTFE_50, PTFE_50_N200, PTFE_50_N400 (^#^ *p* < 0.05).

Sample	PVDF	PTFE	PLLA
control	91.1 ± 3.2	117.5 ± 0.7	76.1 ± 8.6
50	102.4 ± 21.3	82.8 ± 8.7 ^&^	82.8 ± 8.7
50_N200	98.3 ± 12.5	113.6 ± 5.4	82.1 ± 6.7
50_N400	83.3 ± 16.6	109.3 ± 5.1	77.3 ± 11.6
50_O200	87.9 ± 4.6	106.2 ± 5.4	71 ± 5.4
50_O400	70.2 ± 9.0 *	96.3 ± 10.4 ^#^	53.7 ± 5.7 ^&^

**Table 2:** Poly(vinylidene fluoride (PVDF) chemical composition as resulted from XPS measurements for each surface variant.

	PVDF_Control	PVDF_50	PVDF_50_N200	PVDF_50_N400	PVDF_50_O200	PVDF_50_O400
**Elements (%)**						
C	51.9	57.0	66.1	73.1	68.0	69.6
F	47.4	36.4	23.4	4.7	13.7	7.3
O	0.8	6.6	10.5	14.0	18.3	23.2
N	-	-	-	8.2	-	-
**C bonds (%)**						
-CF_2-_	39.8	32.3	10.3	-	8.0	-
-CH_2_-	51.8	41.0	42.7	15.7	20.4	16.5
-C-OH-	3.5	2.8	8.6	4.0	9.6	13.4
-C=O-	-	-	-	24.6	-	22.3
-C-C-	4.9	23.9	38.4	55.8	62.0	47.8

**Table 3:** Poly(tetrafluoroethylene) (PTFE) chemical composition as resulted from XPS measurements for each surface variant.

	PTFE_Control	PTFE_50	PTFE_50_N200	PTFE_50_N400	PTFE_50_O200	PTFE_50_O400
**Elements (%)**						
C	32.2	30.4	29.9	28.8	29.8	31.4
F	66.8	67.7	68.1	69.2	66.6	67.2
O	1.0	1.9	2.0	2.0	3.6	1.4
N	-	-	-	-	-	-
**C bonds (%)**						
-CF_2-_	95.0	79.5	68.8	49.4	71.5	77.1
-CF	-	11.2	9.4	32.2	9.7	12.2
-C-OH-	-	4.2	11.2	6.9	7.3	-
-C-C-	5.0	5.1	10.6	11.5	11.5	10.7

**Table 4:** Poly-L-(lactic acid) (PLLA) chemical composition as resulted from XPS measurements for each surface variant.

	PLLA_Control	PLLA_50	PLLA_50_N200	PLLA_50_N400	PLLA_50_O200	PLLA_50_O400
**Elements (%)**						
C	67.1	74.8	76.2	71.7	75.6	75.3
O	32.9	25.2	23.8	28.3	24.4	24.7
N	-	-	-	-	-	-
**C bonds (%)**						
–C–C–	53.5	69.4	68.0	60.9	72.0	73.7
–C–O–	23.4	19.3	21.0	21.1	17.1	17.4
–O=C–	23.1	11.3	11.0	17.0	10.9	8.9
**O bonds (%)**						
–O=C–	55.6	63.0	62.9	60.1	62.7	61.6
–O–C–	44.4	37.0	37.1	39.9	37.3	38.4

**Table 5:** Modification parameters for studied samples.

Polymer	Number of EUV Pulses	Reactive Gas	Valve Opening Time (ms)	Sample Symbol
PVDF	0	None	0	PVDF_control
	50	None	0	PVDF_50
	50	Nitrogen	200	PVDF_50_N200
	50	Nitrogen	400	PVDF_50_N400
	50	Oxygen	200	PVDF_50_O200
	50	Oxygen	400	PVDF_50_O400
PTFE	0	None	0	PTFE_control
	50	None	0	PTFE_50
	50	Nitrogen	200	PTFE_50_N200
	50	Nitrogen	400	PTFE_50_N400
	50	Oxygen	200	PTFE_50_O200
	50	Oxygen	400	PTFE_50_O400
PLLA	0	None	0	PLLA_control
	50	None	0	PLLA_50
	50	Nitrogen	200	PLLA_50_N200
	50	Nitrogen	400	PLLA_50_N400
	50	Oxygen	200	PLLA_50_O200
	50	Oxygen	400	PLLA_50_O400
